# Evidence for developmental vascular-associated necroptosis and its contribution to venous-lymphatic endothelial differentiation

**DOI:** 10.3389/fcell.2023.1229788

**Published:** 2023-07-27

**Authors:** Han Meng, Youyi Zhao, Yuqian Li, Hong Fan, Xuyang Yi, Xinyu Meng, Pengfei Wang, Fanfan Fu, Shengxi Wu, Yazhou Wang

**Affiliations:** ^1^ Department of Neurobiology and Institute of Neurosciences, School of Basic Medicine, Fourth Military Medical University, Xi’an, Shaanxi, China; ^2^ State Key Laboratory of Military Stomatology, National Clinical Research Center for Oral Diseases and Shaanxi Engineering Research, Center for Dental Materials and Advanced Manufacture, Department of Anethesiology, School of Stomatology, Fourth Military Medical University, Xi’an, Shaanxi, China; ^3^ Department of Neurology, The Second Affiliated Hospital of Xi’an Jiaotong University, Xi’an, Shaanxi Province, China

**Keywords:** necroptosis, development, venous-lymphatic system, mlkl, edema

## Abstract

During development, apoptosis removes redundant cells and ensures proper organ morphogenesis. Necrosis is long known as an adult-bound inflammatory and pathologic cell death. Whether there exists physiological necrosis during early development has been speculated but yet clearly demonstrated. Here, we report evidence of necroptosis, a type of programmed necrosis, specifically in perivascular cells of cerebral cortex and skin at the early stage of development. Phosphorylated Mixed Lineage Kinase Domain-Like protein (MLKL), a key molecule in executing necroptosis, co-expressed with blood endothelial marker CD31 and venous-lymphatic progenitor marker Sox18. Depletion of *Mlkl* did not affect the formation of blood vessel network but increased the differentiation of venous-lymphatic lineage cells in postnatal cerebral cortex and skin. Consistently, significant enhancement of cerebrospinal fluid diffusion and lymphatic drainage was found in brain and skin of *Mlkl*-deficient mice. Under hypobaric hypoxia induced cerebral edema and inflammation induced skin edema, *Mlkl* mutation significantly attenuated brain-blood-barrier damage and edema formation. Our data, for the first time, demonstrated the presence of physiological vascular-associated necroptosis and its potential involvement in the development of venous-lymphatic vessels.

## 1 Introduction

Development is a process elaborately regulated by cell proliferation, differentiation, migration and cell death. For long time, it is generally regarded that apoptosis is the only type of programmed cell death during development, which plays important roles in removing redundant cells and shaping organ morphogenesis (Meier et al., 2000). However, early studies on the mutation of key apoptotic molecules such as caspase-8 and FADD, have documented embryonic lethality, indicating that non-apoptotic cell death might be initiated if apoptosis was blocked (Kaiser et al., 2011; Newton et al., 2019).

Necrosis is another major type of cell death which is usually thought as uncontrollable and injury/pathogen induced. Whether there exists physiological necrosis during development is still an open question. Recently, a type of programmed necrosis, namely, necroptosis is identified in various degenerative or inflammatory diseases, such as Alzheimer’s’ disease, spinal cord injury and virus infection (Fan et al., 2016; Weinlich et al., 2017; Verdonck et al., 2022). Mechanistically, necroptosis is triggered by extracellular cell death inducing or inflammatory factors which activate intracellular signaling cascade of receptor-interacting protein kinase-1/3 (RIPK1/3)/mixed lineage kinase domain-like protein (MLKL) (Newton, 2015). RIPK1 and RIPK3 forms a complex and phosphorylates MLKL, leading to transportation of oligomerized p-MLKL to cell membrane and cell death (Cai et al., 2014).

Interestingly, the long known embryonic lethality led by mutation of key apoptotic molecules caspase-8, FADD, or FLIP was found rescued by mutation of key necroptotic molecules, such as RIPK3 (Kaiser et al., 2011; Oberst et al., 2011). These studies suggested that necroptosis might occur in development if apoptosis were inhibited. As the rescuing effects were mainly observed at certain developmental time points (E10.5, E16.5, and P1), necroptosis was proposed as a checkpoint mechanism for aborting fetuses with severe developmental abnormalities (Dillon et al., 2016; Shan et al., 2018). However, whether there were physiological necroptosis during normal development is still unknown.

We speculate that if there were physiological necroptosis, it should take place in multiple organs and might be related to immune development as the function of necroptosis in adult is closely associated inflammation. In present study, we investigated this question in brain and skin and our data demonstrated a vascular associated necroptosis at the early developmental stage of cerebral cortex and epidermis, and further revealed a potential role of necroptosis in the development of venous-lymphatic vessels.

## 2 Materials and methods

### 2.1 Animals

C57BL/6J mice were bought from the animal facility of the Fourth Military Medical University. *Mlkl* ^*−/−*^ mice were a kind gift from Prof. Jiahuai Han as described (Wu et al., 2013). All mice were maintained in specific pathogen-free and housed under 12 light/12 dark cycle, controlled temperature (22°C–24°C) with free access to water and standard rodent chaw. All animal experiments were carried out according to the protocols and animal ethics approved by the Animal Care and Use Committees of Fourth Military Medical University (KY20194067).

### 2.2 Immunofluorescence and Western blotting

For immunofluorescent staining, animals were sacrificed and perfusion fixed. Frozen sections (10–20 μm in thickness) were prepared. After blocking, primary antibodies were incubated overnight as the followings: rabbit anti-p-MLKL antibody (1 : 250,ab196436, Abcam, RRID:AB_2687465), rat anti-CD31 antibody (1 : 400,550274, BD Pharmingen, RRID:AB_393571), goat anti-PDGFRβ antibody (1 : 400,AF1042, RD system, RRID:AB_2162633), rabbit anti-Prox1 antibody (1 : 150,ab199359, Abcam, RRID:AB_2868427), mouse anti-Sox18 antibody (1 : 400,ac-166026, Santa Cruz Biotechnology, RRID:AB_2195662), rabbit anti-COUP-TFII antibody, (1 : 200,ab211777,Abcam, RRID:AB_2895604). After washing, sections were incubated with secondary antibodies conjugated with Alexa Fluor 594 (donkey anti-rabbit 711-585-152 or anti-mouse 715-585-151 IgG, 1 : 800, Molecular probes) or Alexa Fluor 488 (donkey anti-goat IgG, 705-545-147; anti-rat IgG, 712-545-153; or anti-rabbit IgG, 711-545-152, Molecular probes) for 2–4 h at room temperature. The nuclei were stained by DAPI (1: 2000, Sigma D9542).

For *in vivo* propidium iodide (PI) labeling, 5 μL PI (500 μL/mL, P8080, Solarbio) was injected subcutaneously and samples were collected 30 min later.

For Western blotting, tissues were homogenized in RIPA lysis buffer. Protein concentration was measured by BCA assay. Proteins were separated by SDS-PAGE and transferred to PVDF membrane. After blocking, membranes were incubated with anti-MLKL (1 : 1000,37705, Cell Signaling Technology, RRID:AB_2799118), anti-pMLKL (1 : 900,ab196436, Abcam, RRID:AB_2687465) or anti-β-actin (1 : 5000,66009-1-Ig, Proteintech, RRID:AB_2687938) overnight at 4°C. Then, membranes were incubated with HRP-conjugated anti-rabbit or anti-mouse (1:5000; SA00001-1, SA00001-2, Proteintech, RRID:AB_2722565, RRID:AB_2722565) for 1 h at room temperature. Bands were visualized with an ECL kit (Cat#:32106, Thermo).

### 2.3 Electron microscopic study

The embryos were directly dissected. The brains were quickly removed and fixed with 2% glutaraldehyde. P1 pups were perfused with 2% glutaraldehyde. All the tissues were post-fixed in 2% glutaraldehyde for 4 h. Sections (50 μm in thickness) were prepared with a vibratome and fixed again with 1% osmium tetroxide. Then, sections were subsequently dehydrated with graded ethanol and embedded in Epon 812. Ultrathin sections were cut by using an LKB Nova Ultratome (Bromma). Final counterstaining was performed with uranyl acetate and lead citrate. After that, sections were observed, and images were taken by using a JEM-1230 electron microscope (JEM, Tokyo).

### 2.4 CSF tracing

After anesthesia, mice were fixed on a stereotaxic apparatus (68018, RWD), which was connected with a circulating pump. 10 μL Alexa Fluor™-488 tagged cadaverine (1 μg/μL, A30676, Thermofisher) was injected into cerebellar medullary cistern. Mice were sacrificed 30 min following cadaverine injection. Coronal sections were made and images were taken by a whole slide scanning system (VS.200, Olympus). The area of cadaverine diffusion was measured by ImageJ. Diffusion index was calculated by the ratio of tracer diffused area/section area.

### 2.5 Hypobaric hypoxia-induced brain edema

Hypobaric hypoxia was used to induce brain edema as described (Wang et al., 2022). Mice were kept in a 65 cm × 45 cm × 45 cm chamber, which can simulate different altitudes. The altitude was raised to 5,000 m altitude from sea level at a speed of 5 m/s and stayed for 72 h. Then, the mice descended to sea level at a speed of 5 m/sec. 30 min after reaching the sea level, the mice were euthanized, Evens Blue staining and water content measurement were conducted as the following.

### 2.6 Evens Blue staining and water content measurement

For Evens Blue staining, 4% Evens Blue (200 μL/20 g, E808783, MACKLIN) was injected via tail vein injection immediately following hypobaric hypoxia. 30 min later, mice were perfused and brain was dissected. Images were taken by a microscope (Olympus DP70) under bright field. Brain water content was calculated as: (wet weight-dry weight)/wet weight.

### 2.7 Ear lymphatic drainage assay

The lymphatic vessel drainage function in skin was assessed as described with modification (Karaman et al., 2015). Briefly, mice were anesthetized, and 3 μL Alexa Fluor™-488 tagged cadaverine (0.25 μg/μL, A30676, Thermofisher) was injected into the ears with a 29-G insulin syringe (Teramu, Somerset, NJ). The fluorescence image was taken by a microscope (Olympus DP70) immediately or 8 h after cadaverine injection. The fading index was calculated as: (area of 0 h fluorescence-area of 8 h fluorescence)/area of 0 h fluorescence.

### 2.8 CFA-induced paw edema

Twenty microliter CFA (F5881, Sigma) was injected into the plantar surface of a hind paw. Six hour later, the maximum thickness of the paw was measured by using a vernier caliper. Edema index was calculated as (thickness of edema-thickness of control)/thickness of control.

### 2.9 Realtime RT-PCR

RNA was extracted from E10.5 brain and P1 skin using Trizol reagent (Thermo Fisher Scientific, 15596018). Reverse transcription was carried out by using PrimeScript™ RT Master Mix (TaKaRa, RR036A). PCR was performed using TB Green^®^ Premix Ex Taq™ II (TaKaRa, RR820). The ^ΔΔ^Ct method was used to make comparisons among different experimental groups. The primer sequences are as following: TNFα: GCA​CCA​CCA​TCA​AGG​ACT​CA, TGC​ACC​TCA​GGG​AAG​AAT​CTG. IL-1β: TGG​ACC​TTC​CAG​GAT​GAG​GAC​A, GTT​CAT​CTC​GGA​GCC​TGT​AGT​G. GAPDH: CCC​TTA​AGA​GGG​ATG​CTG​CC, TAC​GGC​CAA​ATC​CGT​TCA​CA.

### 2.10 Statistical analysis

The WT and KO mice from same littermates were adopted for comparation. All the morphological analysis was made by experienced researchers who were blind to the experimental design. Data were expressed as mean ± SEM. Double-stained cells from four to six sections in each mouse were pooled for quantification and analyzed by using GraphPad Prism 8.0 and SPSS 21.0 software. Two-tailed unpaired *t*-test and One-way ANOVA were adopted. The normality of data distribution was evaluated by Shapiro-Wilk test. Statistical significance was assessed at levels of *p* < 0.05.

## 3 Results

### 3.1 Vascular-associated necroptosis during the development of cerebral cortex and epidermis

**FIGURE 1:**
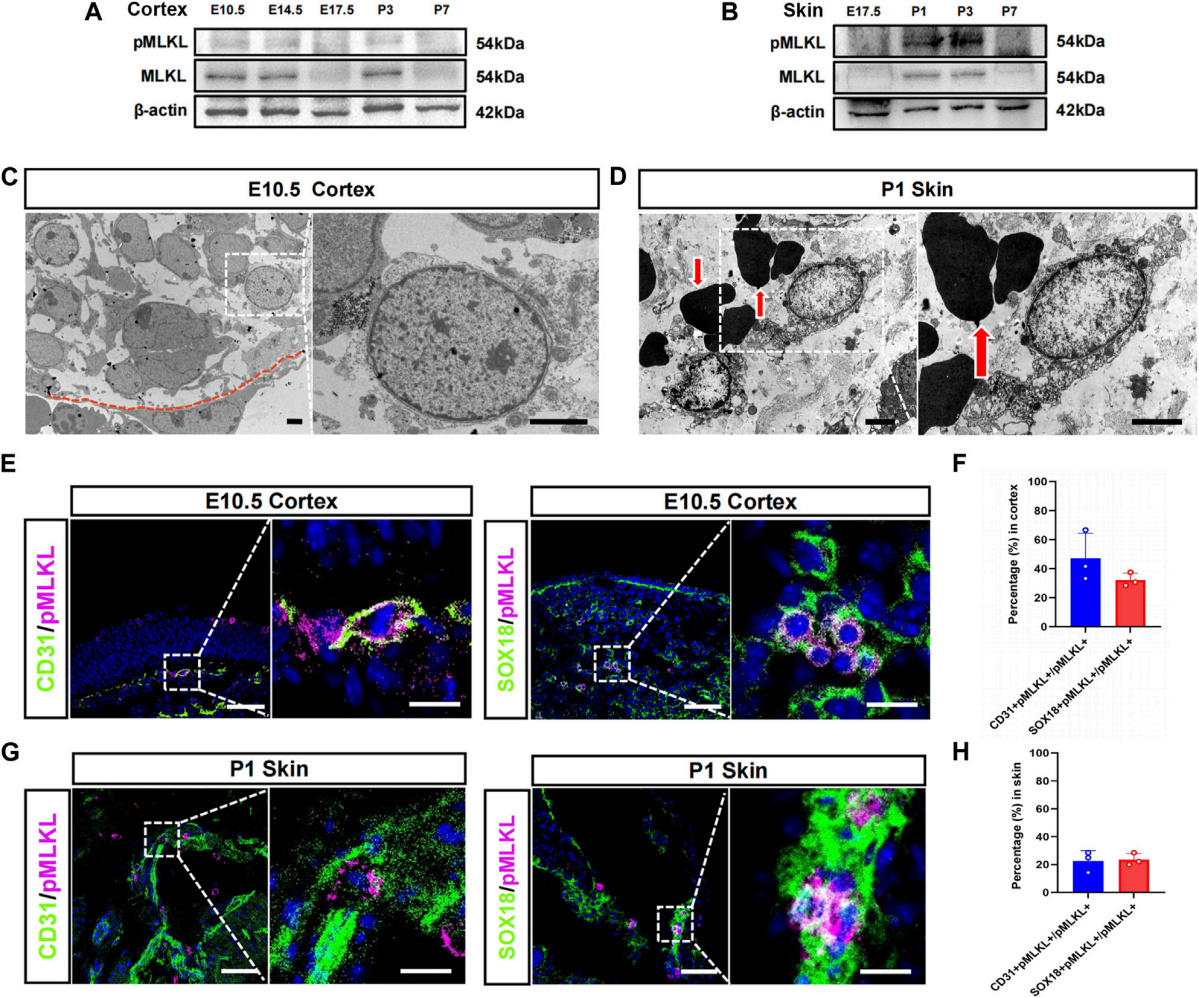
Vascular associated necroptosis in the early developmental stage of cerebral cortex and epidermis **(A, B)** Western blotting of pMLKL and MLKL in brain and skin at different developmental time points **(C)** Representative EM image of necroptotic cells in cortex. Right panel, magnified image. Below the red line is a blood vessel endothelial cell **(D)** Representative EM image of necroptotic cells in skin. Right panel, magnified image. Arrows point to erythrocytes **(E)** Double-immunostaining of CD31/pMLKL and Sox18/pMLKL in cortex **(F)** Percentages of CD31/pMLKL- and Sox18/pMLKL-positive cells in pMLKL-positive cells in cortex **(G)** Double-immunostaining of CD31/pMLKL and Sox18/pMLKL in skin **(H)** Percentages of CD31/pMLKL- and Sox18/pMLKL-positive cells in pMLKL-positive cells in skin. Mean ± SEM. Every one dot in each chart represents the average data of 20–30 cells from one mouse. *n* = 3 mice per group. Bars = 2 μm in **(C, D)**, 40 μm in **(E, G)**, 10 μm in magnified image of **(E)** and **(G)**.

We speculate that if there were physiological necroptosis, it would appear in multiple organs and related to immune development since its function in adult is inflammatory. Tissue-specific macrophages are tissue-resident immune cells which migrated out from blood vessels at specific developmental time points. The developmental time course of the tissue resident macrophage in brain and skin has been well studied, we thus focused on cerebral cortex and epidermis in the present study. Western blotting showed that MLKL and pMLKL appeared to express at E10.5 in brain and at P1 in skin respectively (Figures 1A, B), coinciding with the time point of microglia/Langerhans cell migration (Tamoutounour et al., 2013; Nayak et al., 2014). Under EM, although not very frequently (8 necroptotic cells from 3 copper mesh), we did detect typical necroptotic cells (lysis of cytoplasmic content, disruption of cell membrane and relatively normal nuclear morphology) in cerebral cortex at E10.5 (Figure 1C). In skin (P1), necroptotic cells were easily observed under EM (22 necroptotic cells from 2 copper mesh, Figure 1D). Double-staining of PI with pMLKL confirmed necroptosis in skin (Supplementary Figure S1). Interestingly, all of the necroptotic cells (both in cerebral cortex and in skin) localized close to blood vessel (Figures 1C, D). Particularly, erythrocytes were frequently found adjacent to necroptotic cells in skin (Figure 1D).

To clarify the identity of necroptotic cells, we performed double-immunostaining of p-MLKL with CD31 (endothelial cell marker), PDGFRβ (pericyte marker) and Sox18 (a venous-lymphatic endothelial marker). In cerebral cortex, p-MLKL-positive cells localized mainly at the surface or the interface between cortex and ventricle (Figure 1E). Approximately, 45.5% of the p-MLKL were CD31-positive and 31.4% were Sox18-positive in cerebral cortex (Figure 1F). In skin, p-MLKL-positive cells distributed mainly in epidermal layer (Figure 1G). Approximately 22%–23% of the p-MLKL expressed CD31 or Sox18 (Figure 1H). No detectable co-expression between p-MLKL with PDGFR-β were found in both brain and skin (Supplementary Figure S2). These data demonstrated an occurrence of vascular associated necroptosis during the development of cerebral cortex and skin.

Given that necroptosis is usually associated with inflammation, we examined the expression of TNFα and IL-1β in brain at E10.5, and in skin at P1. Realtime RT-PCR showed no difference of the mRNA of *TNFα* and *IL-1β* between WT and *Mlkl* ^*−/−*^ mice (Supplementary Figure S4).

### 3.2 Dispensable role of necroptosis for blood-vessel formation

To elucidate the function of this developmental necroptosis, we first explored the effects of depleting necroptosis on the tissue residency of microglia and Langerhans cells. As MLKL is the key molecule in executing necroptosis, we adopted *Mlkl* mutant mice for phenotype investigation. The depletion of MLKL was validated by Western blotting (Supplementary Figure S4). Immunostaining of Iba-1 (marker of microglia) showed no significant difference of microglia in the brain of wild type (WT) and *Mlkl* ^*−/−*^ mice (Supplementary Figure S5A, B). Immunostaining of Langerin revealed a mild decrease of Langerhans cells in *Mlkl* ^*−/−*^ skin (Supplementary Figure S5C, D).

**FIGURE 2:**
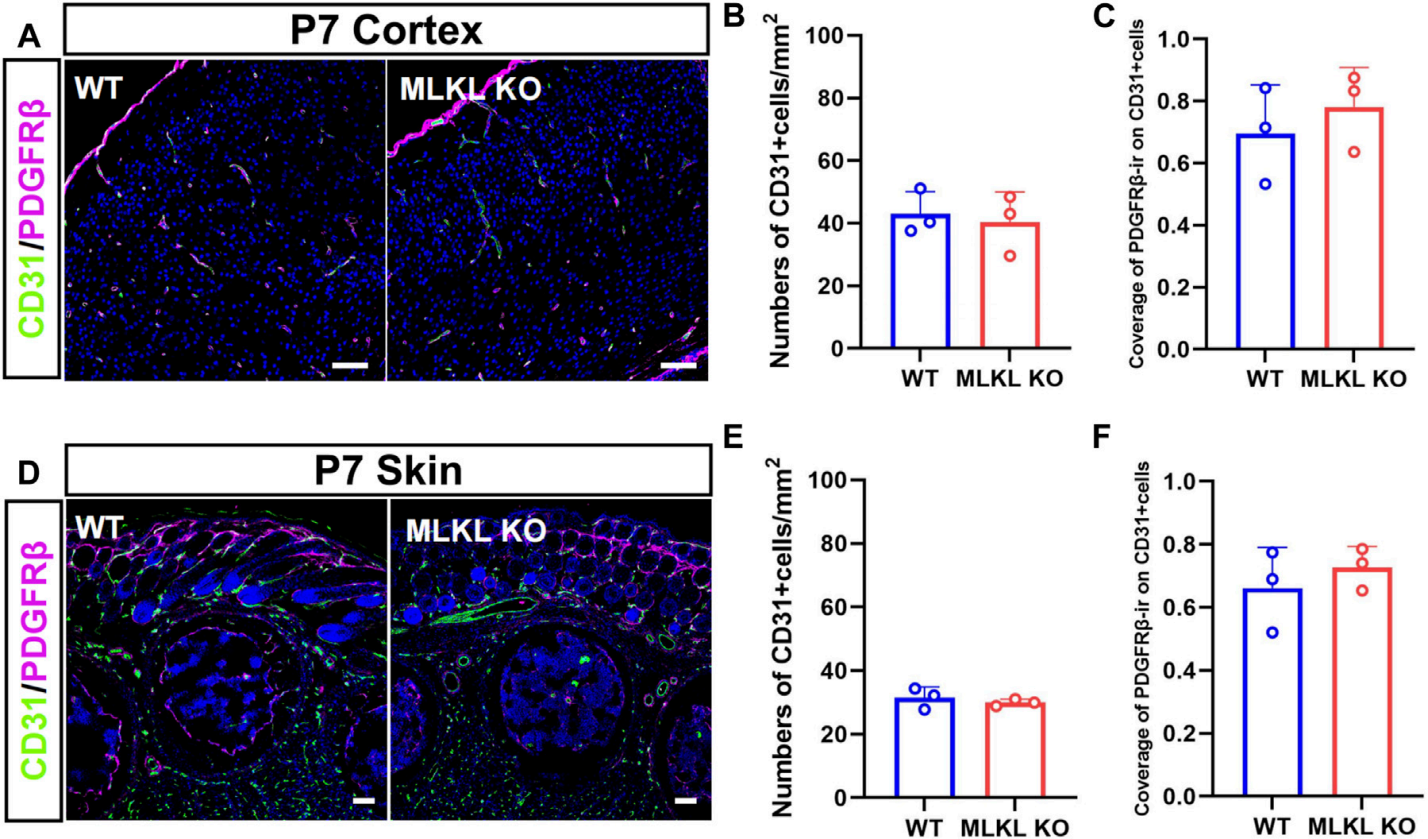
No changes of overall blood vessel formation in the brain and skin of *Mlkl* ^*−/−*^ mice **(A)** Double-immunostaining of CD31/PDGFRβ in cerebral cortex of WT and *Mlkl* ^*−/−*^ mice at P7 **(B, C)** Quantification of CD31-positive cells and coverage of PDGFRβ-ir on CD31-positive cells in **(A) (D)** Double-immunostaining of CD31/PDGFRβ in the skin of WT and *Mlkl* ^*−/−*^ mice at P7 **(E, F)** Quantification of CD31-positive cells and coverage of PDGFRβ-ir on CD31-positive cells in **(D)**. Student’*t*-test. Mean ± SEM. Every one dot in each chart represents the average data of 80–100 cells from one mouse. *n* = 3 mice per group. Bars = 40 μm.

Considering the co-expression between CD31 with pMLKL, we next evaluated if blocking necroptosis would affect vascular development. Double-immunostaining of CD31/PDGFRβ showed no significant change of CD31-positive cells in both cerebral cortex and skin between *Mlkl* ^*−/−*^ mice and WT mice (Figures 2A, B, D, E). Because the interaction between pericyte with endothelial cells were critical for the maturation of blood vessels and the formation of blood-brain barrier (Armulik et al., 2005; Langen et al., 2019), we analyzed the coverage of immunoreactivity of PDGFRβ (PDGFRβ-ir) on CD31-positive cells. The results showed no difference of PDGFRβ-ir/CD31-ir between *Mlkl* ^*−/−*^ mice and WT mice (Figures 2C, F). These data indicated that inhibiting necroptosis did not influence the overall blood-vessel formation in brain and skin.

### 3.3 Increased differentiation of venous-lymphatic endothelial cells in the cerebral cortex and epidermis of *Mlkl* deficient mice

**FIGURE 3:**
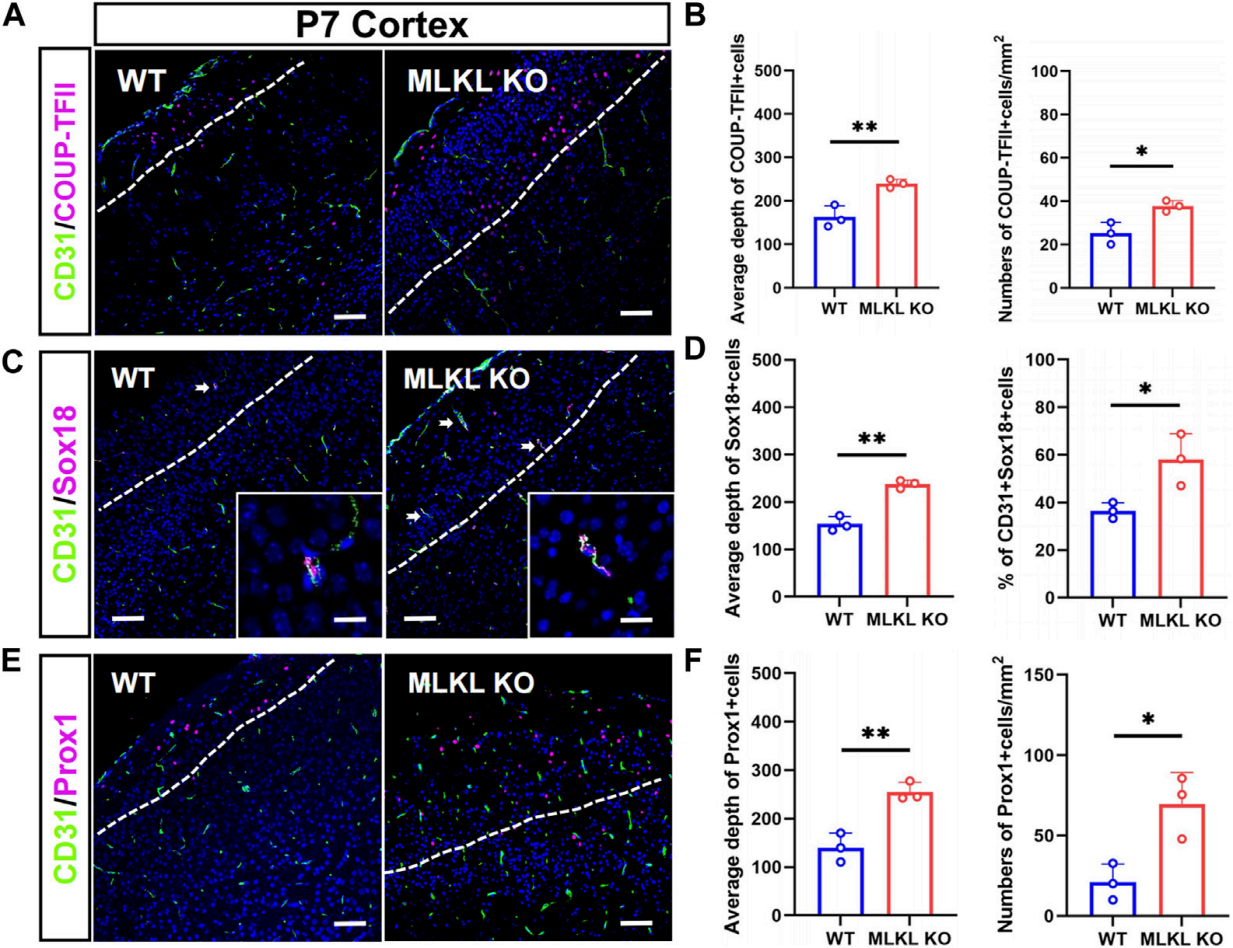
Increased differentiation of venous-lymphatic endothelial cells in the cerebral cortex of *Mlkl* ^*−/−*^ mice **(A, B)** Double-immunostaining of CD31/COUP-TFII and its quantification in cerebral cortex of WT and *Mlkl* ^*−/−*^ mice at P7. Notice the deeper distribution of COUP-TFII -positive cells in *Mlkl* ^*−/−*^ cortex **(C, D)** Double-immunostaining of CD31/Sox18 and its quantification in cerebral cortex of WT and *Mlkl* ^*−/−*^ mice. Notice that the percentages of CD31/Sox18-positive cells increased significantly in *Mlkl* ^*−/−*^ cortex **(E, F)** Double-immunostaining of CD31/Prox1 and its quantification in cerebral cortex of WT and *Mlkl* ^*−/−*^ mice. Notice the deeper distribution of Prox1-positive cells in *Mlkl* ^*−/−*^ cortex. Bars = 40 μm in **(A, C, E)** and 2.5 μm in magnified images. Student’*t*-test. Mean ± SEM. Every one dot in each chart represents the average data of 80–90 cells from one mouse. *p < 0.05. **p < 0.01. *n* = 3 mice per group. White lines indicate the depth of COUP-TFII/Sox18/Prox1-positive cell distribution.

We next assessed if *MLKL* mutation could alter the development of venous-lymphatic lineage cells. In brain and skin, the glymphatic/lymphatic vessels become functional mature around P7-P14 (Karpanen et al., 2006; Munk et al., 2019). We conducted double-immunostaining of CD31 with COUP-TFII (a key transcription factor for determining the fate of vein endothelial cells), Sox18, and Prox1 (a lymphatic transcription factor downstream of Sox18) at P7. In WT mice, COUP-TFII-positive cells localized at the upper layers of cerebral cortex (Figure 3A), consisting with the distribution of glymphatic vessels. In *Mlkl* ^*−/−*^ cortex, a much deeper distribution and bigger number of COUP-TFII-positive cells were observed (Figure 3B). Similarly, Sox18 exhibited a deeper distribution from the surface of cerebral cortex and skin of *Mlkl* ^*−/−*^ mice, as compared with that in WT mice (Figures 3C–F). Particularly, a higher percent of CD31/Sox18-positive cells were found in *Mlkl* ^*−/−*^ cortex (Figure 3D).

**FIGURE 4:**
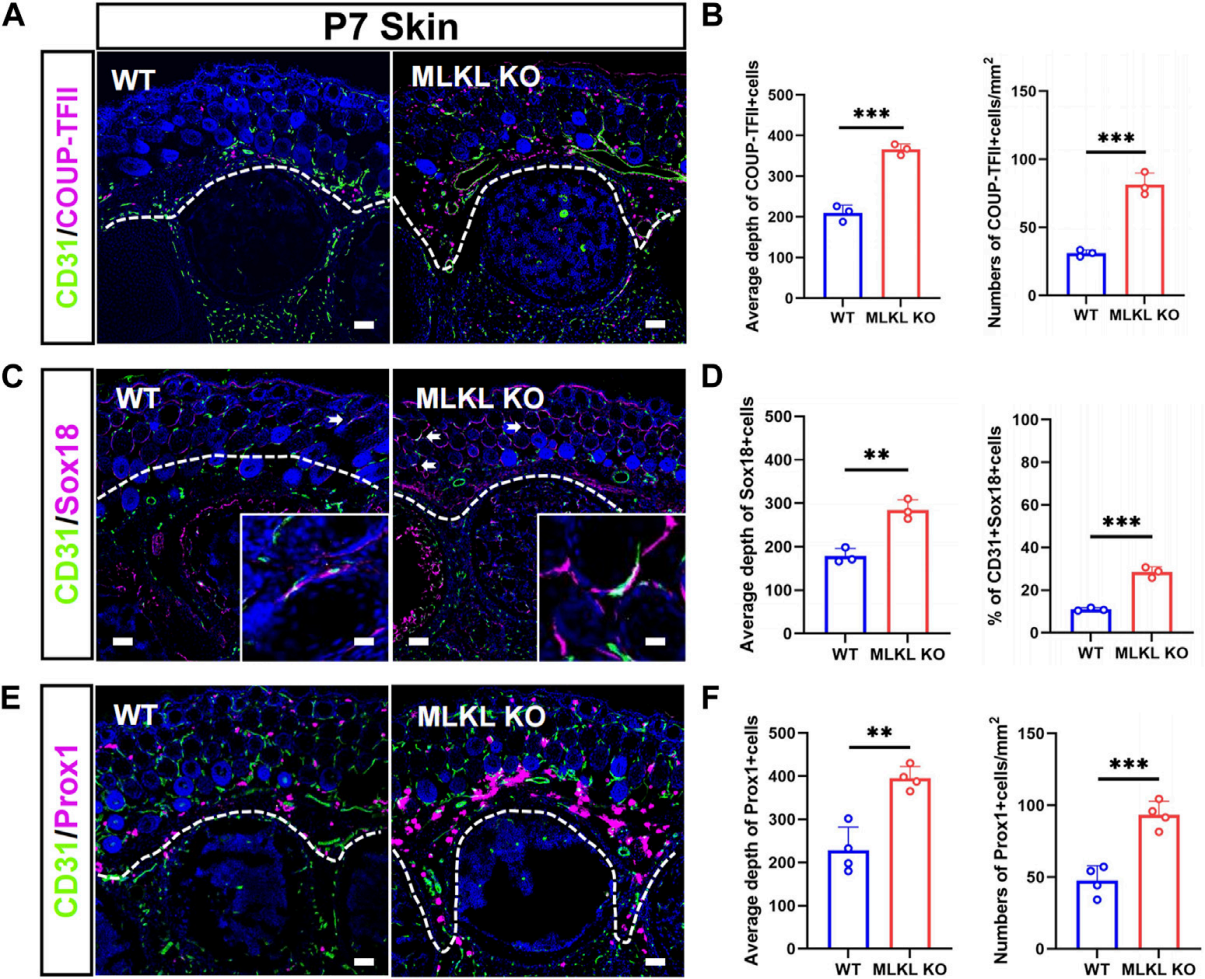
Increased differentiation of venous-lymphatic endothelial cells in the epidermis of *Mlkl* ^*−/−*^ mice **(A, B)** Double-immunostaining of CD31/COUP-TFII and its quantification in the skin of WT and *Mlkl* ^*−/−*^ mice at P7. Notice the deeper distribution of COUP-TFII -positive cells in *Mlkl* ^*−/−*^ skin **(C, D)** Double-immunostaining of CD31/Sox18 and its quantification in the skin of WT and *Mlkl* ^*−/−*^ mice. Notice that the percentages of CD31/Sox18-positive cells increased significantly around hair follicle in *Mlkl* ^*−/−*^ epidermis **(E, F)** Double-immunostaining of CD31/Prox1 and its quantification in the skin of WT and *Mlkl* ^*−/−*^ mice. Notice the deeper distribution of Prox1-positive cells in *Mlkl* ^*−/−*^ skin. Bars = 40 μm. Student’*t*-test. Mean ± SEM. **p < 0.01. ***p < 0.001. *n* = 3 mice per group in **(A–D)**, 4 mice per group in **(E, F)**. Bars = 40 μm in **(A, C, E)** and 2.5 μm in magnified images. Every one dot in each chart represents the average data of 90–100 cells from one mouse. White lines indicate the depth of COUP-TFII/Sox18/Prox1-positive cell distribution.

In the skin of WT mice, COUP-TFII-, Sox18-and Prox1-positive cells localized mainly in the epidermal layer (Figures 4A, C, E). In *Mlkl* ^*−/−*^ mice, COUP-TFII-, Sox18-and Prox1-positive cells expanded beyond epidermal layer and even into mesenchyme layers (Figures 4B, D, F). Quantification showed more COUP-TFII-, Sox18/CD31-and Prox1-positive cells in the epidermal of *Mlkl* ^*−/−*^ mice (Figures 4B, D, F). These data indicated that *Mlkl* mutation led to an increase of the differentiation of venous-lymphatic endothelial cells in the cerebral cortex and epidermis.

### 3.4 Enhanced venous-lymphatic drainage in brain and skin of *Mlkl* deficient mice

**FIGURE 5:**
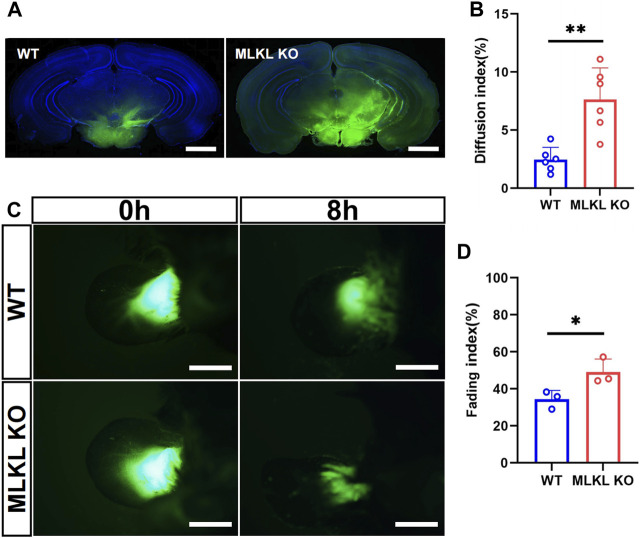
Enhanced lymphatic drainage in the brain and skin of *MLKL* ^*−/−*^ mice **(A)** Representative image of CSF tracing in the brain of WT and *Mlkl* ^*−/−*^ mice under normal condition **(B)** Quantification of fluorescence diffusion **(C)** Representative image of lymphatic drainage in the ear of WT and *Mlkl* ^*−/−*^ mice under normal condition **(D)** Quantification of fluorescence fading. Bars = 1 mm in **(A)**, 500 μm in **(C)**. Student’*t*-test. Mean ± SEM. Every one dot represents the diffusion index or fading index of one mouse. *p < 0.05. **p < 0.01. *n* = 6 mice per group in **(A, B)**, 3 mice per group in **(C, D)**.

We next explored if the deletion of *Mlkl* affected venous-lymphatic drainage in brain and skin. In brain, there is no typical lymphatic vessels. The analog is named as glymphatic system (Cao et al., 2022), which is a network of tunnel-like perivascular spaces allowing interstitial fluid clearance and waste removal through the flow of cerebral spinal fluid (CSF) (Rasmussen et al., 2018; Bohr et al., 2022). We adopted Alexa Fluor™-488 tagged cadaverine to trace CSF flow (Zhang et al., 2018). *Mlkl* ^*−/−*^ mice exhibited remarkably larger area of tracer diffusion in the same time as compared with that in WT mice (Figures 5A, B). To evaluate lymphatic drainage in skin, we injected Alexa Fluor™-488 tagged cadaverine into ear as described (Karaman et al., 2015), and measured the fading of fluorescence. The area of cadaverine fluorescence in *Mlkl* ^*−/−*^ mice shrank much quicker than that in WT mice (Figures 5C, D). These data indicated that *Mlkl* ^*−/−*^ mice had a more robust lymphatic drainage function.

**FIGURE 6:**
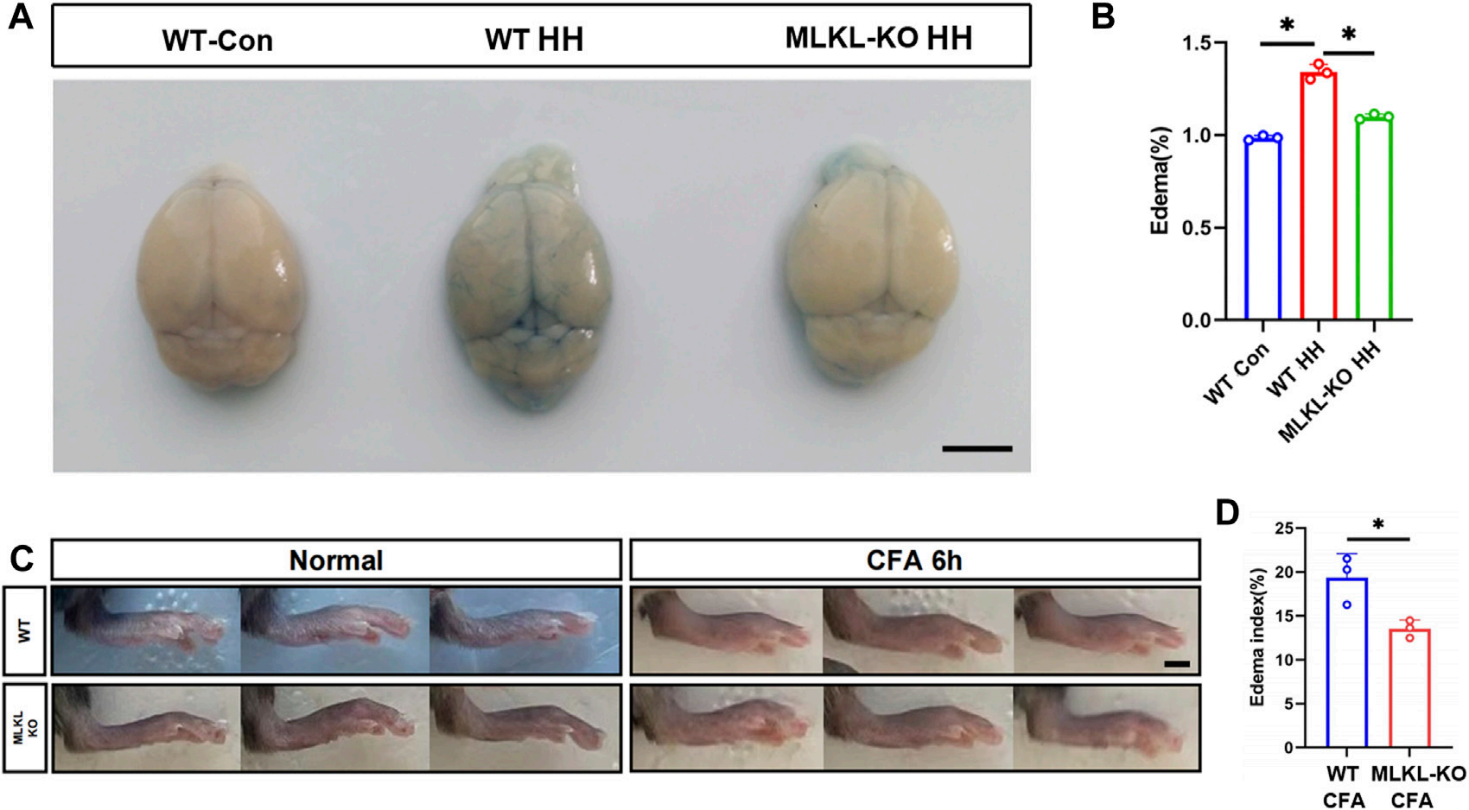
Reduced edema formation in the brain and skin of *Mlkl* ^*−/−*^ mice **(A)** Evens Blue staining in WT mice (WT-Con), WT mice exposed to Hypobaric hypoxia (WT-HH), *Mlkl* ^*−/−*^ mice exposed to Hypobaric hypoxia (*Mlkl*-KO HH). Notice the reduced Evens-Blue staining in *Mlkl*-KO HH **(B)** Water content in WT-Con, WT-HH, *Mlkl*-HH. Notice the recovery of water content in *Mlkl*-KO HH **(C)** Representative images of foot edema in WT and *Mlkl* ^*−/−*^ mice injected with or without CFA **(D)** Edema index of WT or *Mlkl*-KO mice injected with CFA. One-way ANOVA in **(B)**. Student’*t*-test in **(D)**. Mean ± SEM. Every one dot represents the edema index of one mouse. *p < 0.05. *n* = 3 mice per group. Bar = 2 mm.

To validate this observation, we evaluated edema formation in brain by exposing mice to hypobaric hypoxia. Evens-Blue staining was used to assess the integrity of blood-brain barrier. Extensive Evens-Blue leakage was observed in WT mice after 3days’s hypobaric hypoxia (HH) treatment. In contrast, very weak Evens-blue staining was detected in the cerebral cortex of *Mlkl* ^*−/−*^ mice (Figure 6A). In line with this, water content measurement showed that *Mlkl* deficiency dramatically compromised the water increase induced by HH (Figure 6B). In skin, we assessed local edema formation induced by CFA injection. Six hours post CFA injection, the maximum paw thickness in *Mlkl* ^*−/−*^ mice was significantly thinner than that in WT mice (Figures 6C, D). These data supported the observation that *Mlkl* deficiency enhanced lymphatic drainage.

## 4 Discussion

In the present study, we first provided EM evidence of vascular-associated necroptosis in cerebral cortex and epidermis at their early stage of development. By examining the biomarkers expression, we demonstrated a robust differentiation of venous-lymphatic endothelial cells at neonatal stage of *Mlkl* mutant mice. Further, our data revealed that *Mlkl* deficient mice bear much rapid lymphatic drainage under both normal and edematous conditions.

Early studies have reported non-apoptotic death of certain cells during embryonic development of *drosophila*, *C. elegance* and vertebrates (Borsello et al., 2002; Abraham et al., 2007; Mondragon et al., 2019). In those studies, non-apoptotic cell death was identified by ultrastructural morphology, but not defined as necrosis. The rescuing effects of depleting necroptotic genes in *fadd-* or *caspase-8*-deficiency mice shed new light on the possibility of developmental necroptosis (Green et al., 2011; Oberst et al., 2011). So far as we known, all these developmental necroptosis were found under the conditions of apoptosis inhibition. Our data, for the first time, provided evidence of physiological necroptosis. Interestingly, necroptotic cells localized specifically around blood vessels, suggesting this necroptosis is well-regulated rather than spontaneously occurs. In the present study, we reported necroptosis in brain and skin. Whether there exists necroptosis in other tissues remains to be explored.

In adult tissues, necroptosis is mainly induced by cell death signals, such as TNFα. One interesting question is how necroptosis is triggered during development. It is possible that transient expression of some cytokines or death-associated molecular pattern may trigger necroptosis. Alternatively, formation of Z-DNA may activate ZBP1-dependent necroptosis (Jiao et al., 2020).

The most important finding of present study, in our eyes, is the contribution of necroptosis to venous-lymphatic endothelial cell differentiation. At the early stage of lymphatic development, lymphatic endothelial progenitor cells detach from vein endothelial chain and start differentiation (Marziano et al., 2021). It is reasonable that some cells may die during this process. Our finding that *Mlkl* mutation significantly increased the invasion of COUP-TFII-, Sox18-and Prox1-positive cells, enhanced lymphatic drainage, but did not affect CD31-positive endothelial cells, demonstrated a novel role of necroptosis in directly regulating the differentiation of lymphatic endothelial cells. As MLKL bears certain non-apoptotic functions, such as regulating lipid accumulation and release of extracellular vesicles (Yoon et al., 2017; Rasheed et al., 2020; Zhan et al., 2021), the data do not exclude the possibility that MLKL directly regulates lymphatic endothelial differentiation. One weak point of present study is not using conditioned knockout mice. Mechanistic and lineage tracing studies on how necroptotic cells regulate the development of venous-lymphatic vessels are worthy to be conducted in the future.

Why does evolution reserve necroptosis specifically in lymphatic vessel development? Neonatal pups are usually subjected to a world fraught with pathogens. At this stage, precociously functional lymphatic drainage would possibly lead to wide spread of infected virus. Therefore, developmental necroptosis may serve as a protective mechanism for the survival of organisms by preventing the over formation of immature lymphatic vessels at neonatal stage. Nevertheless, our data implied that inhibiting developmental necroptosis may be beneficial for reducing edema.

## Data Availability

The original contributions presented in the study are included in the article/Supplementary Material, further inquiries can be directed to the corresponding authors.
