# Supplementary material for: Evidence for developmental vascular-associated necroptosis and its contribution to venous-lymphatic endothelial differentiation
Source: Front Cell Dev Biol. 2023 Jul 27;11:1229788. doi: 10.3389/fcell.2023.1229788 (PMC10416103; doi:10.3389/fcell.2023.1229788)
Supplement: Supplementary file 1 [file DataSheet1.docx]

Supplementary Material

Evidence for developmental vascular-associated necroptosis and its contribution to venous-lymphatic endothelial differentiation

Han Meng^1#^, Youyi Zhao^2#^, Yuqian Li^1#^, Hong Fan^3#^, Xuyang Yi^1^, Xinyu Meng^1^, Pengfei Wang^1^, Fanfan Fu^1^, Shengxi Wu^1^*, Yazhou Wang^1*^

*** Correspondence:** Prof. Yazhou Wang, yazhouw@fmmu.edu.cn; Prof. Shengxi Wu, [shengxi@fmmu.edu.cn](mailto:shengxi@fmmu.edu.cn)

# Supplementary Figures


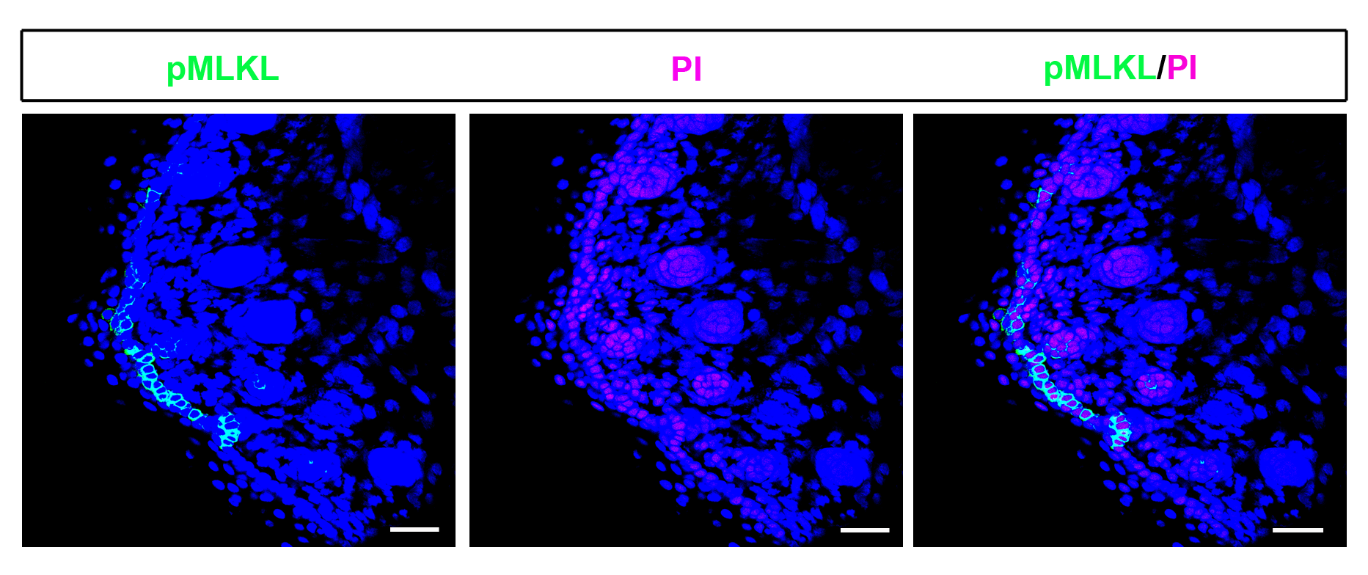


**Supplementary Figure 1.** Combined *in vivo* PI-labeling with immunostaining of pMLKL at P1. Notice the PI/pMLKL-positive cells. Bar = 50 μm.

**
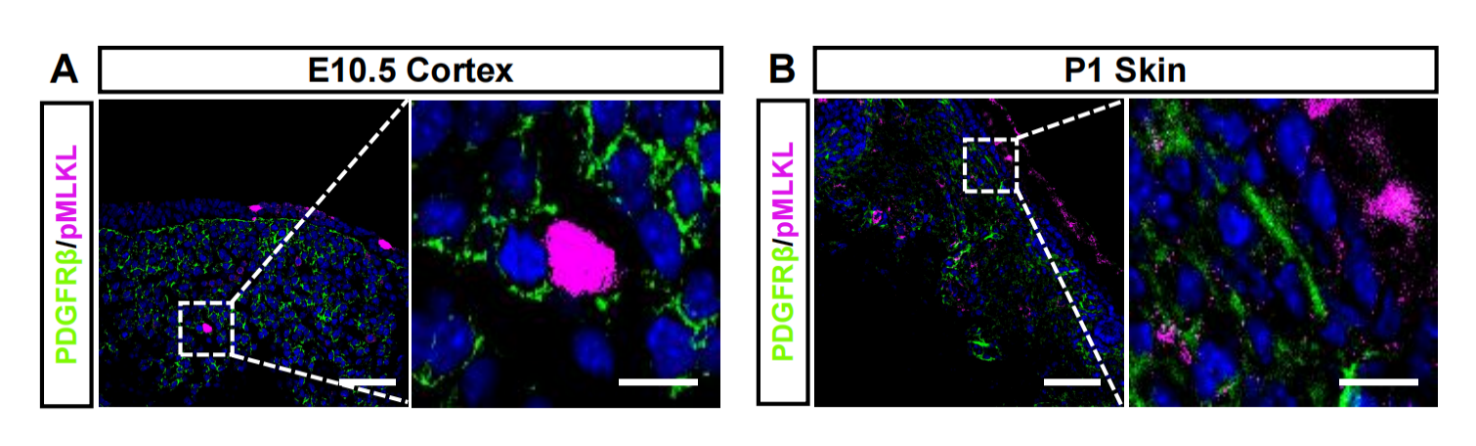
**

**Supplementary Figure 2.** Double immunostaining of pMLKL with PDGFRβ in brain at E10.5 and in skin at P1. No co-expression of pMLKL with PDGFRβ was observed. Bars = 40 μm and 10 μm in magnified images.


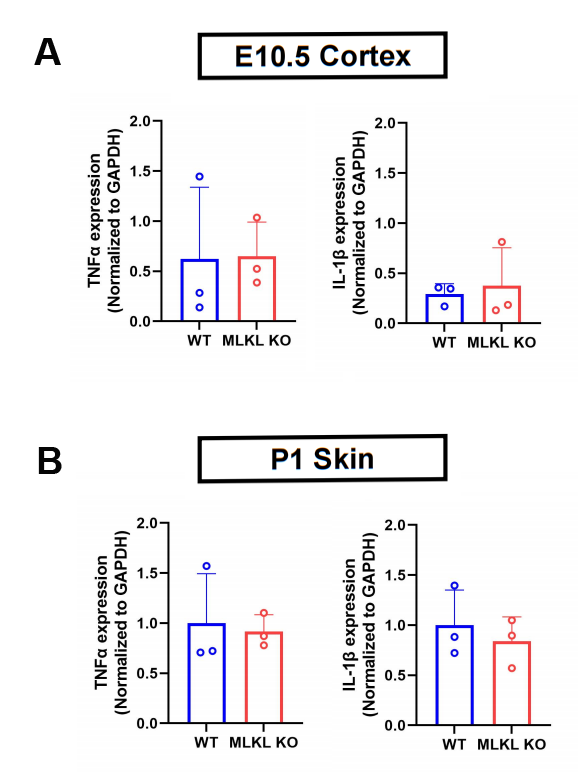


**Supplementary Figure 3.** Real-time RT-PCR of TNFα and IL-1β in E10.5 brain and P1 skin.


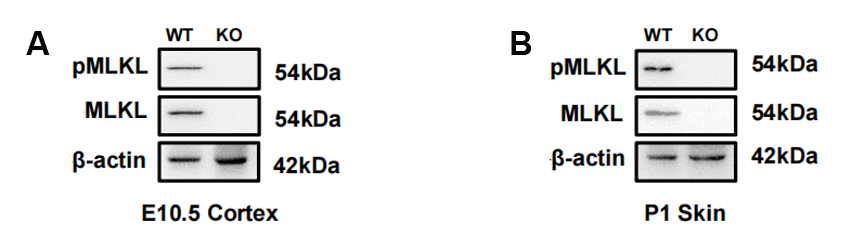


**Supplementary Figure 4.** Western-blotting of pMLKL and MLKL in cortex and skin of WT and *Mlkl^-/-^* (KO) mice.


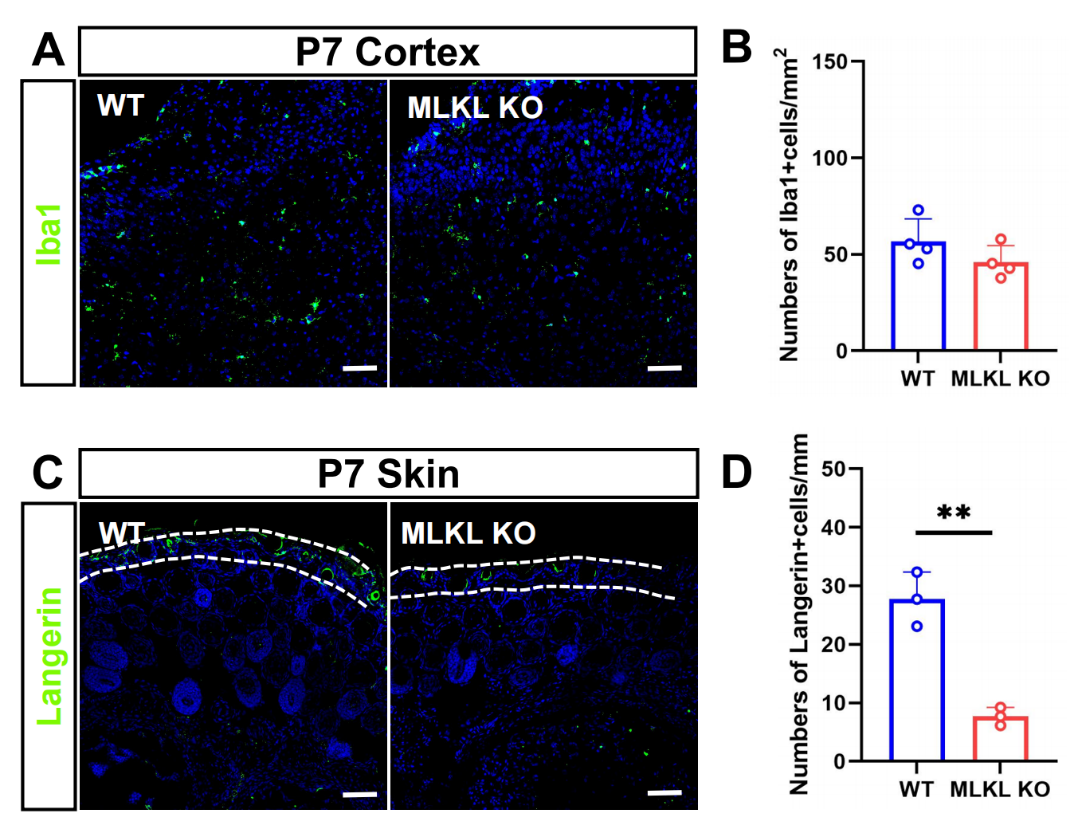


**Supplementary Figure 5.** (A, B) Immunostaining and quantification of Iba-1 in cortex of WT and *MLKL^-/-^* mice at P7. (C, D) Immunostaining and quantification of Langerin in skin of WT and *MLKL^-/-^* mice at P7. Bars = 40 μm. Student’t test. ***P*<0.01.
